# 1031-1034delTAAC (Leu125Stop): a novel familial *UBE3A* mutation causing Angelman syndrome in two siblings showing distinct phenotypes

**DOI:** 10.1186/1471-2350-13-124

**Published:** 2012-12-20

**Authors:** Greice Andreotti De Molfetta, Cristiane Ayres Ferreira, Daniel Onofre Vidal, Liane de Rosso Giuliani, Maria José Maldonado, Wilson Araujo Silva

**Affiliations:** 1 Department of Genetics, Medical School of Ribeirao Preto, University of Sao Paulo, Sao Paulo, Brazil; 2 Regional Blood Center of Ribeirao Preto and National Institute of Science and Technology in Cell Therapy, Ribeirao Preto, Brazil; 3 Department of Pediatrics, Federal University of Mato Grosso do Sul, Campo Grande, Brazil; 4 CTC – Centro de Terapia Celular da Fundação Hemocentro de Ribeirão Preto, Laboratório de Genética Molecular e Bioinformática, Depto. De Genética, Rua Tenente Catão Roxo, 2501, 14051-140, Ribeirão Preto, São Paulo, Brazil

**Keywords:** Angelman syndrome, *UBE3A* gene, Imprinting, Novel mutation, Distinct phenotypes, HRM

## Abstract

**Background:**

More than 50 mutations in the *UBE3A* gene (E6-AP ubiquitin protein ligase gene) have been found in Angelman syndrome patients with no deletion, no uniparental disomy, and no imprinting defect.

**Case Presentation:**

We here describe a novel *UBE3A* frameshift mutation in two siblings who have inherited it from their asymptomatic mother. Despite carrying the same *UBE3A* mutation, the proband shows a more severe phenotype whereas his sister shows a milder phenotype presenting the typical AS features.

**Conclusions:**

We hypothesized that the mutation Leu125Stop causes both severe and milder phenotypes. Potential mechanisms include: i) maybe the proband has an additional problem (genetic or environmental) besides the *UBE3A* mutation; ii) since the two siblings have different fathers, the *UBE3A* mutation is interacting with a different genetic variant in the proband that, by itself, does not cause problems but in combination with the *UBE3A* mutation causes the severe phenotype; iii) this *UBE3A* mutation alone can cause either typical AS or the severe clinical picture seen in the proband.

## Background

Angelman syndrome (AS) is a neuro-genetic disorder characterized by intellectual and developmental delay, sleep disturbance, seizures, jerky movements, frequent laughter or smiling and a happy disposition [1]. The incidence of AS is estimated to be between 1/10,000 and 1/20,000 [2] and is inherited in an autosomic dominant trait, modified by imprinting, or inherited by imprinting [3]. Analysis of parent-specific DNA methylation pattern in the 15q11-13 chromosome region detects approximately 77% of individuals with AS, including those cases with a deletion (approximately 70%), uniparental disomy (1-2%), or an imprinting defect (3-5%); fewer than 1% of individuals have a cytogenetically chromosome rearrangement and *UBE3A* sequencing detects mutations in approximately 5-10% of the patients [4]. In 10-15% of the cases the molecular exam is normal with no deletions, uniparental disomy, imprinting defects or *UBE3A* mutations [5]. Recently, it was demonstrated that the Angelman syndrome protein Ube3A is a neuronal activity-regulated protein that controls synaptic function by ubiquitinating and degrading the synaptic protein Arc. In the absence of Ube3A, elevated levels of Arc accumulate in neurons resulting in the excessive internalization of AMPA receptors at synapses and impaired synaptic function [6]. We report a brother and sister who was referred to our laboratory in order to investigate a clinical suspicion of AS. As the analysis of the differential parental specific DNA methylation within the 15q11-13 region was normal, we investigated the *UBE3A* gene in order to screen for mutations causing AS. We here describe a novel *UBE3A* frameshift mutation in two siblings who have inherited it from their asymptomatic mother. Despite carrying the same *UBE3A* mutation, the proband shows a more severe phenotype whereas his sister shows the typical AS features associated to a milder phenotype.

## Case presentation

### Case report

**Figure 1:**
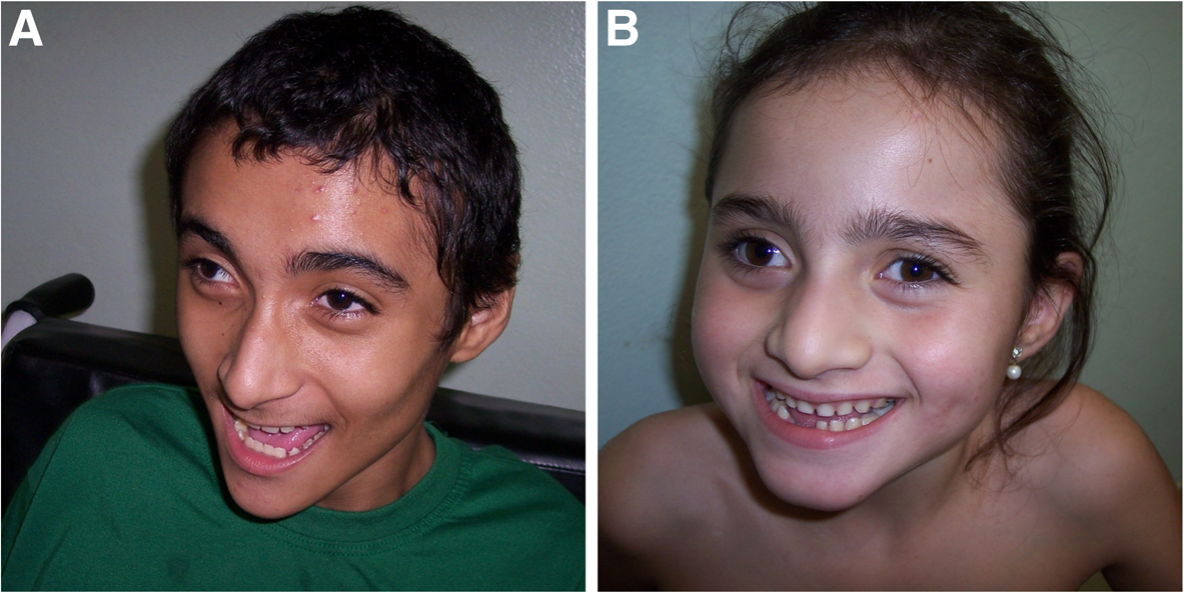
**Patients here reported.** In **A)** we observe the proband clinical features; In **B)** we observe the proband’s sister clinical features.

Patient 1, the proband, aged 14 years and 8 months, was born from non-consanguineous and healthy parents (Figure 1A). After a normal pregnancy, the patient was born at term by cesarean section, weighing 2,900 g (25th-50th centile), length and head circumference were not informed. His developmental progress was delayed; he sat at 1 year old, he is not able to walk. At the age of 9 months, he began to have seizures and has been on valproic acid with good control. Unlike typical AS patients, he did not display a happy disposition with frequent smiling and laughing. He has normal EEG and brain MRI shows reduction of the volume of the inferior cerebellar vermis, increase of the cisterna magna, diffuse atrophy with decreased volume more pronounced in the fronto-temporal region, ventriculomegaly, thin corpus callosum. When he was examined at the age of 11 years old, his head circumference was 47 cm (<3th centile), height and weight were not informed. He presented with normal cytogenetic analysis and normal study of the methylation within the 15q11-13 region.

**Figure 2:**
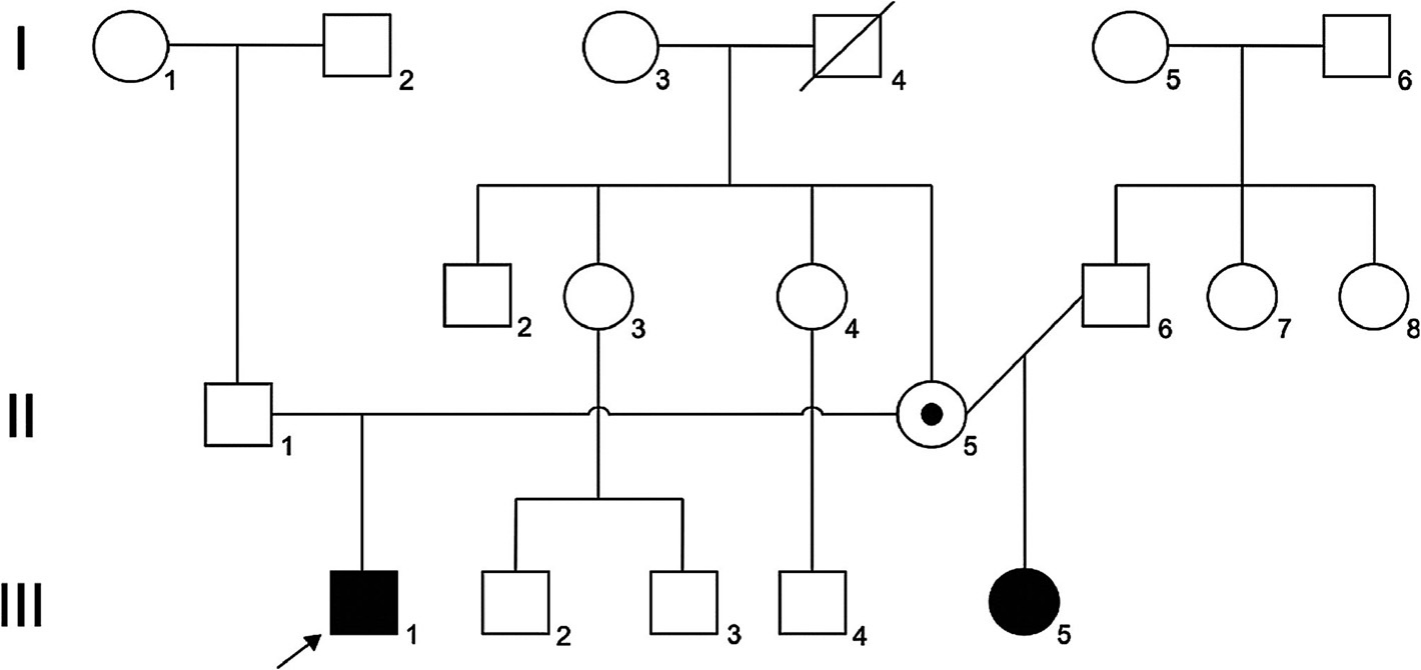
**Histogram representing the pedigree evaluated in the case reported.** Black square: proband (patient 1 – III.1); Black circle: proband’s sister (patient 2 – III.5).

Patient 2, the proband’s sister, aged 6 years and 3 months, was born from non-consanguineous and healthy parents (Figure 1B); these two siblings have different fathers (Figure 2). The prenatal period was uneventful and the baby was delivered by cesarean section at full term with a birth weight of 3385 g (50^th^-75^th^ centile), height of 50 cm (50^th^-75^th^ centile), and head circumference of 35 cm (75^th^-90^th^ centile). Her developmental progress was delayed. She presented hypotonia by age of 7 months, sat at 2 years old, walked at 3 years old. At the age of 3 years old, she began to have seizures and has been on fenobarbital with good control. She has the typical AS phenotype, happy behavior, wide mouth with wide-spaced teeth, uplifted flexed arm position, ataxic gait, and excessive devotion for playing with water. When she was examined at the age of 4 years old, her head circumference was 47.5 cm (<3th centile), height 110 cm (97th centile), and weight 20.000g (95th centile). She presented the typical EEG for AS patients, normal brain MRI, normal cytogenetic analysis and study of the methylation within the 15q11-13 region.

### Methods

#### DNA extraction

The genomic DNA was extracted from leukocytes from peripheral blood samples, using Super Quick-gene-rapid DNA isolation (Promega), following the manufacturer’s instructions.

#### Methylation analysis using Methylation Sensitive High Resolution Melting (MS-HRM) assay

For methylation analysis of the *SNRPN* gene, DNA conversion using bisulfite and cleanup of 2 μg of genomic DNA was carried out using EpiTect Bisulfite Kit (Qiagen), following the manufacturer’s instructions. Afterwards the DNA conversion, each sample was run in duplicate by the MS-HRM assay to determine the methylation pattern within the 15q11-13 region using primers and protocol described by White et al. (2007) [7]. The MS-HRM analysis was performed in a 7500 Fast Real-Time PCR System (Applied Biosystems) using MeltDoctor HRM Master Mix (Applied Biosystems) according manufacturer’s instructions. The PCR reaction conditions were 95°C for 10 minutes, followed by 40 cycles of 95°C for 15 seconds, 58°C for 1 minute and 95°C for 1 minute, followed by the standard melting curve.

#### PCR and mutation screening using High Resolution Melting (HRM) assay

In order to screen for *UBE3A* mutations, we used the primers described by Malzac et al. (1998) [8] and the HRM technique. The HRM analysis was performed in a 7500 Fast Real-Time PCR System (Applied Biosystems) using MeltDoctor HRM Master Mix (Applied Biosystems) according manufacturer’s instructions. Each sample was run in duplicate and approximately 20 ng of genomic DNA was amplified in a total volume of 20 μl containing 5 μM of each primer, deionizated water, and 10μl of MeltDoctor. master mix (Applied Biosystems). The PCR reaction conditions were 95°C for 10 minutes, followed by 40 cycles of 95°C for 15 seconds, 55°C for 1 minute and 95°C for 1 minute, followed by the standard melting curve.

### Cloning and DNA sequencing

DNA fragments amplified by PCR were subjected to direct sequencing in an automatic capillary sequencing system ABI 3130 Genetic Analyser using BigDye^R^ Terminator v3.1 Cycle Sequencing kit, following the manufacturer’s instructions. The sequencing results were analyzed through FinchTV version 1.4.0 (Geospiza Inc. 2004–2006); the sequences obtained were compared with reference from the GenBank (NM_130.839.1). In order to proper characterize the mutation, the same DNA fragments amplified by PCR were cloned into TOPO^R^ vector using TOPO TA cloning (Invitrogen) according to manufacturer’s instructions.

## Conclusions

**Figure 3:**
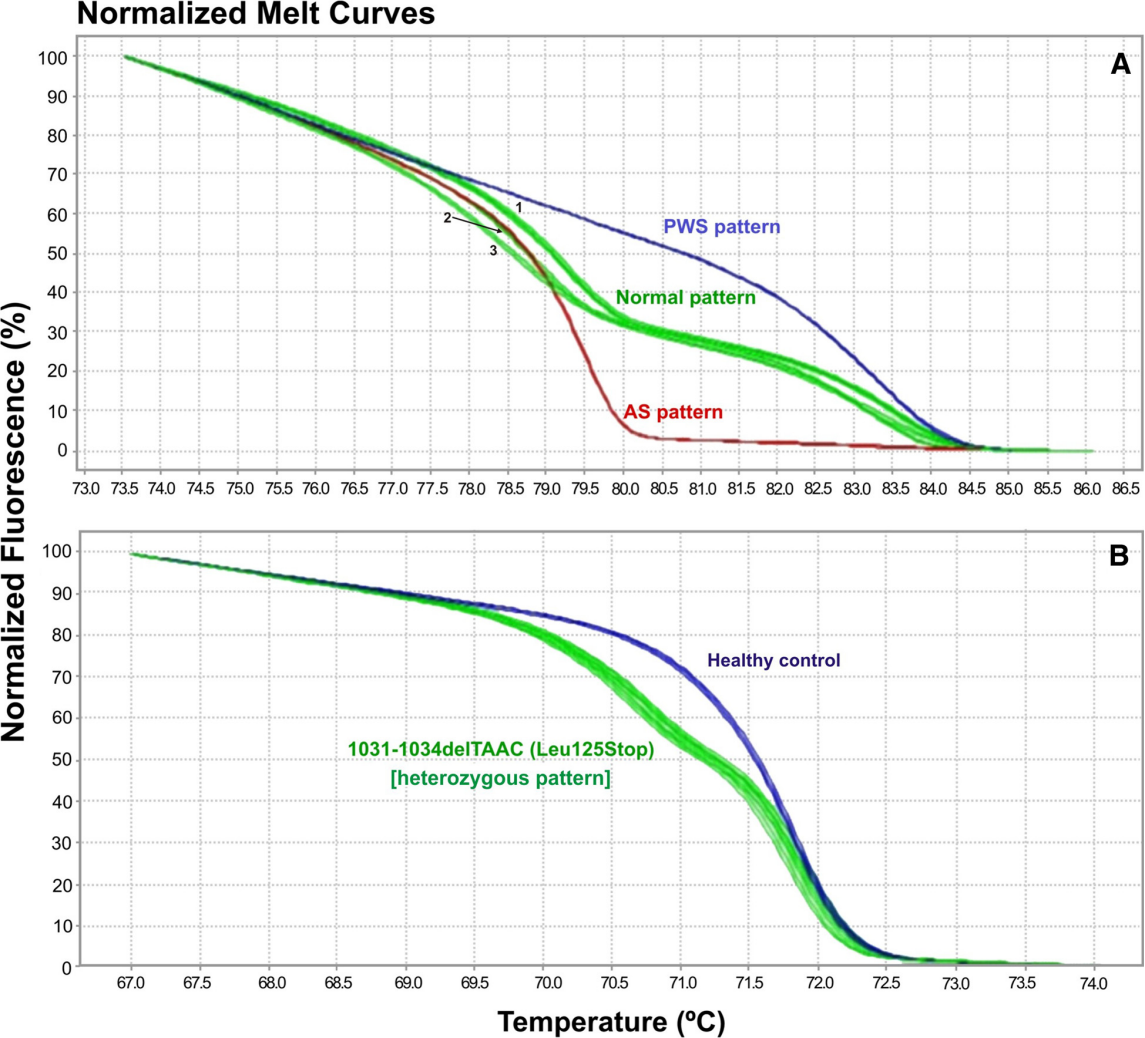
**High resolution melthing analysis.** For both MS-HRM and HRM for screening for mutations, each sample was run in duplicate. In **A)** the melting curves observed for the PCR fragment analyzed in methylation-specific HRM (MS-HRM) assay for the *SNRPN* locus are shown. Blue lines represent the pattern of methylation of *SNRPN* for patients affected by Prader-Willi Syndrome (PWS); Red lines represent the pattern of methylation of *SNRPN* for patients affected by Angelman Syndrome (AS); Green lines represent the normal pattern of methylation of *SNRPN* locus: 1 shows the methylation curve detected in the normal control and mother (II.1); 2 shows the methylation curve detected in patient III.1 and 3 shows the methylation curve detected in patient III.5. In **B)** the melting curves observed for the PCR fragment analyzed in HRM assay for the screening of mutations in the *UBE3A* gene are shown. Blue lines represent the melting curve pattern obtained for healthy controls presenting with a normal *UBE3A* gene (without any mutation); Green lines represent the melting curve pattern obtained for all individuals (II.1, III.1 and III.5) presenting with the 1031-1034delTAAC mutation in the *UBE3A* gene.

**Figure 4:**
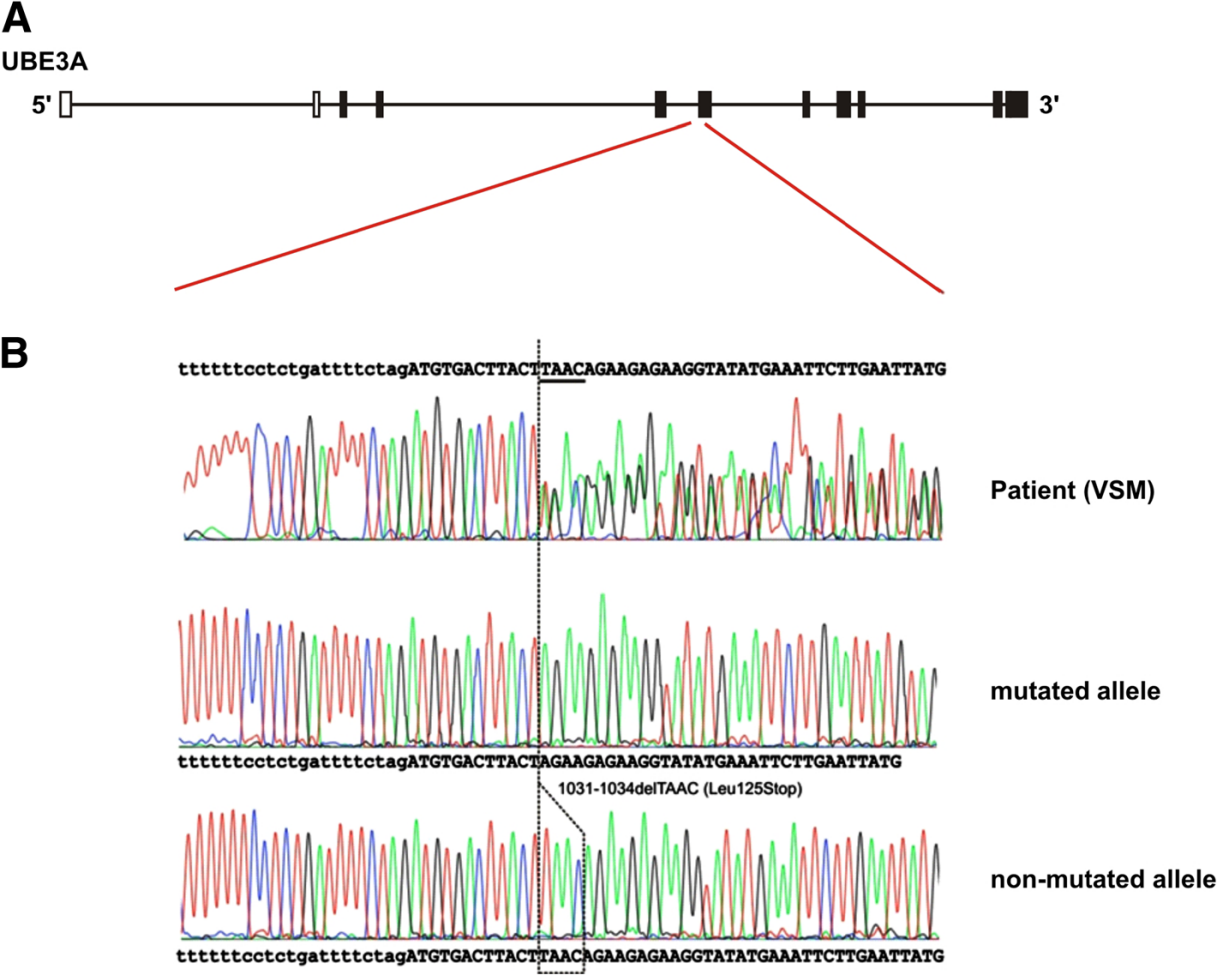
**DNA sequencing of *UBE3A *gene for patients III.1 and III.5. A)** Schematic representation of the structure of *UBE3A* gene. Squares: exons; White squares: untranslated regions (UTRs); Black squares: coding exons; Black line: introns. **B)** The upper panel shows the direct sequencing of the DNA fragment obtained subsequently to PCR. Middle and lower panels show the sequencing of the same DNA fragments amplified by PCR after cloning into TOPO TA cloning vector (Invitrogen). The middle panel shows the sequencing obtained for the mutated allele [1031-1034delTAAC (Leu125Stop)], and the lower panel shows the sequencing obtained for the non-mutated allele.

Kubota et al., (1997) [9] have developed a methylation-specific PCR assay for detection of the PWS and AS based on the methylation status of the CpG island within the SNRPN gene. More recently a similar protocol was developed based on comparing melting profiles of experimental samples to melting profiles from DNA with known methylation levels. This assay is called Methylation Sensitive HRM (MS-HRM) [10] and is also employed to analyze the differential methylation at the SNRPN locus [7]. Carrying on our methylation experiments by using the MS-HRM protocol, analysis of samples from the two siblings with AS phenotype and their asymptomatic mother (Figure 2) revealed a normal methylation pattern (Figure 3A), excluding the possibility of deletion, uniparental disomy or imprinting mutation encompassing the *SNRPN* locus. In order to characterize the molecular defect, the entire coding region of *UBE3A* gene was screened for mutations using the HRM method. The result revealed that exon 9 of both siblings and their mother share an abnormal pattern of melt curve when compared with the control (Figure 3B). Afterwards, the DNA sequencing of exon 9 showed a deletion of four nucleotides (TAAC) affecting codons 125 and 126 resulting in a frameshift mutation, which creates a premature stop codon at position 125, 1031-1034delTAAC (Leu125Stop) (Figure 4). In this scenario, we concluded that the mutation was transmitted from a paternal chromosome inherited from the mother. The majority of mutations found within *UBE3A* gene are localized at the *hect* domain region, which includes the 3′region of the exon 9, extends through the exon–16. All of them are frameshift mutations leading to stop codons [11-15]. The mutation described in the present study is localized at the begining of exon 9 causing a frameshift mutation changing the entire *hect* domain. Huibregtse et al. (1995) [16] have previously reported that a UBE3A mutant protein lacking the last six amino acids is completely defective in ubiquitination. Thus, we predict that the protein product from the mutated UBE3A reported here will also be completely defective in ubiquitination. We report here that in spite of carrying the same mutation, the proband shows a more severe phenotype as he is not able to walk and does not present some of the typical AS facial features as wide mouth with wide-spaced teeth, happy behavior, arm positions and movements. Also, he presents abnormal MRI showing severe abnormalities which are not the typical MRI findings for AS. On the other hand, his sister presents a milder phenotype, compared to her brother, including the typical AS features as wide mouth and spaced teeth, ataxic gait, arm movements, happy behavior, attraction to water. It has already been reported that *UBE3A* mutations resulting in frameshifts and/or premature truncations give a more severe phenotype than missense mutations or short in-frame deletions that still allow the translation of a full-length protein [4]. Baumer et al. (1999) [14] have reported that patients with *UBE3A* mutations and deletions present a more severe phenotype as the patient shows all the clinical features and, the milder phenotype is reported among patients carrying paternal uniparental disomy and imprinting defect. Also, Malzac et al. (1998) [8] reported that the phenotype due to *UBE3A* mutation did not differ regardless of the mutation and they also reported that the clinical phenotype presented by the patient carrying a *UBE3A* mutation is the typical AS phenotype. However, some patients carrying *UBE3A* mutations showed milder phenotype, as they do not present ataxic gait, seizures, microcephaly, and normal EEG [13,15]. We hypothesized that the mutation Leu125Stop causes both severe and milder phenotypes. We reported a similar observation where a duplication of GAGG at exon 10 of *UBE3A* caused different phenotypes in first cousins [17]. Perhaps similar mechanisms explain the phenotypic differences between the related patients. Potential mechanisms include: i) maybe the proband has an additional problem (genetic or environmental) besides the *UBE3A* mutation; ii) since the two siblings have different fathers, the *UBE3A* mutation is interacting with a different genetic variant in the proband that, by itself, does not cause problems but in combination with the *UBE3A* mutation causes the severe phenotype; iii) this *UBE3A* mutation alone can cause either typical AS or the severe clinical picture seen in the proband.

In addition, as shown in Figure 3A, we observed three differential methylation patterns. This result may be associated to the phenotypic variability observed between patients, due to the differential methylation pattern along the SNRPN analyzed region containing 21 CpG dinucleotides. To test this hypothesis we performed the bisulfite sequencing analysis of the patients, their mother and a normal control (Additional file 1). All the samples showed the same heterozygous CpG methylation pattern as expected in cases of AS patients with biparental inheritance (Additional file 1). We also observed that all cytosine in CpG dinucleotides were heterozygous (T/C) with a small T peak height compared to the C peak height. The bisulfite conversion was efficient since all the isolated cytosine was fully converted into thymine. No mutation was observed in the same region. The imbalance observed between the methylated allele (observed as the C peak in the chromatogram) and the non-methylated allele (observed as the T peak in the chromatogram) is due to the peripheral blood cell heterogeneity where CpG methylation status is differentially maintained in the PWS/AS region [18,19].

It is worthy to discuss that this is the first paper using HRM to search for mutations within the *UBE3A* gene. HRM is a relatively new technique, which is being widely used as a method for screening for mutations [20]. Therefore, it provides very simple solutions for genotyping as this technique combines high sensitivity and specificity making it useful for personalized DNA diagnostics.

## Consents

Written informed consent was obtained from the patient’s mother for publication of this Case Report and any accompanying images. A copy of the written consent is available for review by the Series Editor of this journal.

## Competing interests

The authors declare that they have no competing interests.

## Authors’ contributions

GAM: carried out the molecular genetic studies (HRM, MS-HRM, DNA sequencing) and drafted the manuscript. CAF and DOV: carried out the HRM. LRG: evaluated the patients. MJM: carried out the clinical exams such as EEG, MRI. WASJ: have given final approval of the version to be published. All authors read and approved the final manuscript.

## Pre-publication history

The pre-publication history for this paper can be accessed here:

http://www.biomedcentral.com/1471-2350/13/124/prepub

## Supplementary Material

Additional file 1 **DNA sequencing after bisulfite conversion.** A) Shows part of the SNRPN region analyzed in this work. 1. SNRPN sequence before bisulfite conversion; in bold we show the CpG dinucleotides. 2. SNRPN sequence after bisulfite conversion; in red we show the isolated cytosine which were converted into thymine. B) The sequencing obtained for the mother (II.5), patient III.1 and patient III.5 after the bisulfite conversion. * shows the CpG dinucleotides. Click here for file
