# Supplementary figures and images for: 1031-1034delTAAC (Leu125Stop): a novel familial UBE3A mutation causing Angelman syndrome in two siblings showing distinct phenotypes
Source: BMC Med Genet. 2012 Dec 20;13:124. doi: 10.1186/1471-2350-13-124 (PMC3543165; doi:10.1186/1471-2350-13-124)

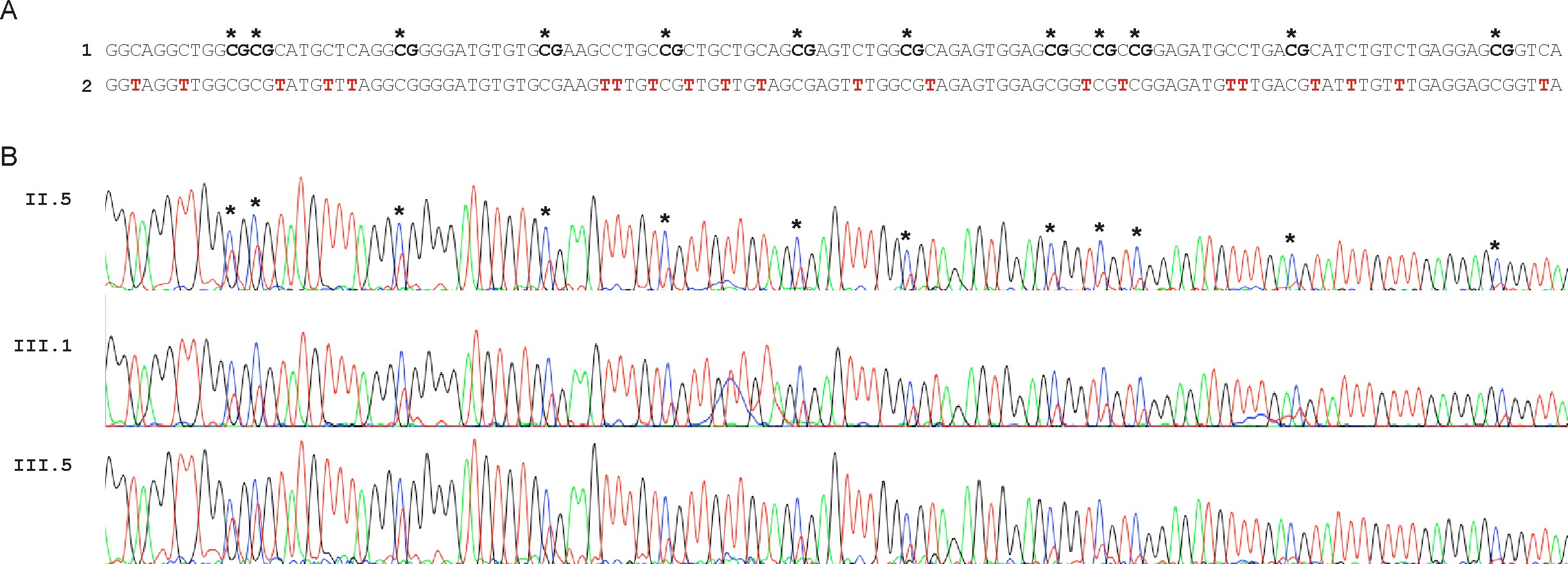

Supplement: Additional file 1 — DNA sequencing after bisulfite conversion. A) Shows part of the SNRPN region analyzed in this work. 1. SNRPN sequence before bisulfite conversion; in bold we show the CpG dinucleotides. 2. SNRPN sequence after bisulfite conversion; in red we show the isolated cytosine which were converted into thymine. B) The sequencing obtained for the mother (II.5), patient III.1 and patient III.5 after the bisulfite conversion. * shows the CpG dinucleotides. [file 1471-2350-13-124-S1.jpeg]
